# The Impact of COVID-19 Disease on Urology Practice

**DOI:** 10.1055/s-0041-1725155

**Published:** 2021-06-03

**Authors:** Mohamad Moussa, Mohamed Abou Chakra, Athanasios G. Papatsoris, Athanasios Dellis

**Affiliations:** 1 Department of Urology, Al Zahraa Hospital & Lebanese University, Beirut, Lebanon; 2 Department of Urology, Lebanese University, Beirut, Lebanon; 3 2nd Department of Urology, School of Medicine, National and Kapodistrian University of Athens, Sismanoglio Hospital, Athens, Greece; 4 Department of Urology/General Surgery, Areteion Hospital, Athens, Greece

**Keywords:** COVID-19, urology practice, oncology, pandemic, endourology, kidney transplant

## Abstract

The diagnosis and timely treatment of cancer patients should not be compromised during an infectious disease pandemic. The pandemic of coronavirus disease 2019 (COVID-19) has serious implications on urology practice and raises particular questions for urologists about the management of different conditions. It was recommended to cancel most of the elective urological surgeries. Urological cancers surgeries that should be prioritized are radical cystectomy for selective tumors, orchiectomy for suspected testicular tumors, nephrectomy for c T3 + , nephroureterectomy for high-grade disease, and radical adrenalectomy for tumors >6 cm or adrenal carcinoma. Most prostatectomies can be delayed without compromising the survival rate of patients. Urological emergencies should be treated adequately even during this pandemic. There is a potential risk of coronavirus diffusion during minimally invasive procedures performed. It is crucial to use specific precautions when urologists performed those type of surgeries. It was also recommended to suspend the kidney transplantation program during the COVID-19 pandemic except for specific cases. In this review, we discussed the triage of urological surgeries, the risk of minimally invasive urological procedure, the kidney transplantation challenges, the systemic therapies, intravesical instillation of Bacillus Calmette-Guérin (BCG), endourology, teleconferencing, and telemedicine application in urology during the COVID-19 pandemic.

In late December 2019, a previous unidentified coronavirus, currently named as the 2019 novel coronavirus, emerged from Wuhan, China, and resulted in a formidable outbreak in many cities in China and expanded worldwide. The disease is officially named as coronavirus disease-2019 (COVID-19). COVID-19 is a potential zoonotic disease with low to moderate (estimated 2–5%) mortality rate. Person-to-person transmission may occur through droplet or contact transmission. 1 Classical public health measures, including isolation, quarantine, social distancing, and community containment, can be used to curb the pandemic of this respiratory disease. Wearing masks, washing hands, and disinfecting surfaces contribute to reducing the risk of infection. Clinical trials are underway to investigate the efficacy of new antiviral drugs, convalescent plasma transfusion, and vaccines. 2 The novel coronavirus uses the same receptor, angiotensin-converting enzyme 2 as that for SARS-CoV, and mainly spreads through the respiratory tract. Importantly, increased evidence showed sustained human-to-human transmission, along with many exported cases across the globe. The clinical symptoms of COVID-19 patients include fever, cough, fatigue, and a small population of patients also showed gastrointestinal infection symptoms. The elderly and people with underlying diseases are susceptible to infection and prone to serious outcomes. 3

To prepare for a pandemic, hospitals need a strategy to manage their space, staff, and supplies so that optimum care is provided to the patient. In the operating room (OR), specific preparations are required. These include engineering controls such as identification and preparation of an isolated OR, administrative measures such as modification of workflow and processes, the introduction of personal protective equipment (PPE) for staff, and formulation of clinical guidelines for anesthetic management. 4 The COVID-19 crisis has resulted in an unprecedented global demand for PPE, with the rapid pandemic escalation leaving limited time for equipment reserve preparation. Severe acute respiratory syndrome coronavirus 2 (SARS-CoV-2) ribonucleic acid (RNA) has also been detected in multiple asymptomatic individuals, some with a similar viral load to symptomatic patients. Potential transmission from asymptomatic persons has also been reported. Intubating a patient with COVID-19 is a high-risk procedure owing to the proximity of the health care workers to the patients' oropharynx and the exposure to airway secretions, which can carry a high viral load. 5

After the WHO declaration, the United States Surgeon General proclaimed a formal advisory to cancel elective surgeries at hospitals due to the concern that elective procedures may contribute to the spreading of the coronavirus. Elective procedures can pragmatically be stratified into “essential,” which implies that there is an increased risk of adverse outcomes by delaying surgical care for an undetermined period, versus “nonessential” which alludes to purely elective procedures that are not time-sensitive for medical reasons. 6 Cutting back on elective procedures at ambulatory centers may be crucial to slowing the spread of SARS-CoV-2, as well as make capacity, staff, and equipment available to address COVID-19 as the outbreak spreads. 7

Changes have occurred in clinical and academic settings across the major urological centers in Europe. During the pandemic, there is a shortage of resources such as bed occupancy and the availability of PPE. There is a negative impact of the pandemic on scientific, academic, and educational activities, as well as on the personal and social life of urologists and other health care providers. 8

The characteristics of an “elective” procedure in urology are case-dependent and have not been well defined in the current pandemic. At the same time, one should aim to ensure adequate care for urological emergencies and urgent urological treatments as far as possible, even during the pandemic. For this, patients must be prioritized individually; alternative therapy concepts must be considered, and regional cooperation must be used. 9 It is crucial to share up-to-date information regarding the best urology practices during the COVID-19 pandemic while maintaining the safety and well-being of all urologists. The review aims to present the currently available literature about urology practice during the COVID-19 pandemic. Also, we will present the role of e-Health and its application in this critical time.

## Triage of Urological Surgeries during COVID-19 Pandemic

Urology practice was affected by the COVID-19 pandemic. In one of the busiest department of urology in Bergamo, Italy, there was a major change in surgical scheduling. This was related to many factors. First, the anesthesiologists need to work with patients with acute COVID-19 disease. Second, the number of beds used for urological patients was reduced. Third, there was intent to not expose urology patients to hospital contamination and fourth, the number of urology ward staff was reduced. 10 Based on those factors elective surgeries were canceled and selection criteria are required to classify surgeries as elective and not elective. A suggested triage of surgical procedures during the COVID-19 pandemic is summarized in Table 1 .

**Table 1:** Triage of urologic surgeries during the COVID-19 pandemic

Surgeries should be performed with high priority	Surgeries should be delayed
1. Radical cystectomy 2. Orchiectomy 3. Nephrectomy with selected criteria. 4. Nephroureterectomy 5. Adrenalectomy 6. Ureteral stent insertion for obstruction. 7. Infected artificial urinary sphincter explant. 8. Infected penile prosthesis explant. 9. Shunting for priapism. 10. Testicular detorsion/orchidopexy. 11. Scrotal abscesses/Fournier's gangrene debridement. 12. Penile/testicular fracture repair. 13. Ureteral injury/bladder perforation. 14. Deceased donor transplant. 15. Surveillance cystoscopy/diagnostic cystoscopy. 16. Post-chemotherapy retroperitoneal residual lymph nodes. 17. Radical prostatectomies in selected cases. 18. Intravesical BCG therapy for selected cases of bladder cancer.	1. Radical prostatectomies in selected cases. 2. BPH procedures. 3. Stress urinary incontinence. 4. Fistula repair. 5. Infertiliy surgeries. 6. Live donor transplants. 7. Peyronie's disease surgeries. 8. Penile prosthesis insertion. 9. Prostate biopsy. 10. Flexible cystoscopy. 11. Intravesical BCG therapy for selected cases of bladder cancer. 12. Inguinal hernia. 13. Circumcision. 14. Surgery for vesicoureteral reflux. 15. Pyeloplasty in UPJ obstruction without loss of differential function. 16. Urolithiasis without infection or obstruction.

Abbreviations: BCG, Bacillus Calmette-Guérin; COVID-19, coronavirus disease 2019; UPJ, uretero-pelvic junction.

Urological oncological diseases and obstructive uropathies will inevitably get priority in times of reduced operating theater availability and outpatient procedures. Patients with nonurgent conditions can be deferred by a few months or prescriptions sent electronically. Where there is a high degree of suspicion for malignancy or obstructive urinary disorders patients can be called into reduced capacity emergency outpatient clinics. 11

For urological tumors, radical cystectomy, orchiectomy, and nephrectomy with selected criteria, nephroureterectomy and adrenalectomy should be prioritized. For endourological procedures, when possible, stents or nephrostomy tubes can be placed at the bedside to spare a ventilator. BPH surgeries can be delayed. For stress urinary incontinence, interstitial cystitis, overactive bladder, neurogenic bladder, all surgeries can be delayed except for nerve simulators removal. It can be done under local anesthesia as outpatient. Fistula repair surgery should be delayed when possible. If there is an infected artificial urinary sphincter explant or infected penile prosthesis, surgery is emergent. For urethral obstruction, all procedures can be delayed. In general urology, shunting for priapism, testicular detorsion/orchidopexy, scrotal abscesses, Fournier's gangrene debridement, penile/testicular fracture repair, ureteral injury, and bladder perforation should be prioritized. For kidney transplant surgery, deceased donor transplants only should proceed without delay, live donor transplants should be delayed. For infertility, all surgeries should be delayed. 12

Pediatric urology has wide variations in practice. The variety of surgical indications that need to be considered for prioritization includes obstructive uropathy and recurrent febrile urinary tract infections (UTIs) putting children at intermediate-term risk of loss of renal function. Only surgeries related to organ viability have been performed since the first week of March 2020 such as testicular torsion, incarcerated inguinal hernia, obstructing ureteral stones, Wilms tumor in the timeframe for surgery after chemotherapy. Postponing cryptorchidism surgery for a few months might not be a problem, but if the pandemic is still raging, the fertility of patients will be affected. 13

For patients with Peyronie's disease, requesting a penile implant, and other patients who request therapy for erectile dysfunction would not be able to access treatment. 14

Diagnostic and surveillance cystoscopy should be performed without any delay. A prostate biopsy can be delayed 3 to 6 months if there are no-high risk factors, if there are high risk factors including PSA more than 20, PSA doubling time less than 6 months, digital exam suggesting clinical T3 disease and/or local or systemic symptoms, it is recommended to obtain magnetic resonance imaging initially; delay biopsy up to 3 months; if performing the biopsy, it is suggested to use the transperineal biopsy, if possible, to minimize infectious risks and fecal exposure. The urodynamic study can be delayed for 3 to 6 months. The exchange of chronic foley or suprapubic catheter can be extended for additional 2 to 4 weeks if there is no history of encrustation or recurrent UTI. Androgen deprivation therapy (ADT) can be delayed for 6 to 8 weeks. 15

Urologists can be requested to perform surgeries on COVID-19 patients, procedures should be performed by experienced surgeons, outside of their learning curve. All urological departments performing nonurgent procedures should reduce the number of beds. 16 The greatest risk of urologist for COVID exposure may come from community transmission, during patient interactions, and/or in the OR. At many institutions, low acuity inpatient consultations are being performed remotely with the explicit goal of simply finding an “outpatient home.” 17 The current COVID-19 pandemic is affecting urology practice at every level. The long-term consequences of this scenario are difficult to estimate, as a long follow-up duration will be required to reveal the effects of delayed diagnosis of conditions requiring urgent urological consultations. As we struggle to maintain oncological priorities, patients with nonurgent oncological needs or benign diseases also deserve attention as in many systems with universal health care, these patients are already subject to long waiting lists before they can be treated. Besides the obvious deterioration of quality of life, many complications such as recurrent infection, bleeding, and bladder stones can occur for those patients. 18 During the swift and drastic process of redeployment, one of the many concerns was the maintenance of urologic services. The false-negative rates of the early COVID-19 tests and the presence of asymptomatic carriers did not allow to create a “COVID-free” space. 19 To recover the urology departments from an extraordinary level of suspended activity, urologists were obliged to prioritize pathologies based on purely clinical criteria, for which tables including the relevance of each pathology within each area of urology are being proposed. 20

The new condition with the COVID-19 pandemic obliges urologists to conform to the guidelines that appear on a daily basis formulated by multidisciplinary surgical groups to manage urological emergencies. We must adapt to the resources available, implementing all biosecurity measures to protect patients and all health personnel who are in charge of patient management. 21 Overall, there was a large consensus among different urological associations/societies regarding the prioritization of most urological procedures, including those in the outpatient setting, urological emergencies, and many inpatient surgeries for both oncological and nononcological conditions. On the contrary, some differences were found regarding specific cancer surgeries potentially due to different prioritization criteria and/or health care contexts. 22

## Uro-oncological Surgeries during COVID-19 Pandemic

Cancer patients appear to have an estimated twofold increased risk of contracting SARS-CoV-2 than the general population. The diagnosis and timely treatment of cancer patients should not be compromised during an infectious disease pandemic. During an infectious disease pandemic, patients may face difficulties, including access to hospitalization and deferred surgeries. 23 During the pandemic, triage decisions require even more interspecialist coordination and communication than usual. Postponing surgery and administering neoadjuvant therapy as a bridge can decrease risk to the patient and preserve health care resources. 24 Suggested criteria to help the selection of uro-oncological surgeries during the COVID-19 pandemic are summarized in Table 2 .

**Table 2:** Details of the high priority oncologic surgeries during the COVID-19 pandemic

High priority surgery	Details
TURB high-risk NMIBC	High-risk NMIBC, 2nd look TURBT
Radical cystectomy	MIBC, unresponsive NMIBC
Radical nephroureterectomy	Nonmetastatic UTUC, high-grade and/or cT1 tumors
Radical nephrectomy	cT2b-4 tumors, advanced RCC with IVC thrombi
Radical orchiectomy	Testicular tumors
Partial/total penectomy	cT3 tumors + inguinal lymphadenectomy

Abbreviations: COVID-19, coronavirus disease 2019; IVC, inferior vena cava; MIBC, muscle-invasive bladder cancer; NMIBC, nonmuscle-invasive bladder cancer; RCC, renal cell carcinoma; TURBT, transurethral resection of bladder tumor; UTUC, upper tract urothelial carcinoma.

For urologic tumors, surgeries that should be prioritized are radical cystectomy for muscle invasive bladder cancer and carcinoma in situ refractory to third-line therapy and transurethral resection of bladder tumor (TURBT) for bladder tumors, orchiectomy for suspected testicular tumors. Nephrectomy for c T3+ including all patient with renal vein and inferior vena cava thrombus should be prioritized. Nephrectomy for cT1 and cT2 should be in general delayed. Nephroureterectomy for high-grade disease and radical adrenalectomy for tumors >6 cm or adrenal carcinoma should be prioritized. Most prostatectomies can be delayed without compromising the survival rate of patients. If the patient had prostate cancer with National Comprehensive Cancer Network high risk, surgeries can be an option if he cannot tolerate radiation therapy. 12

A real-life overview of the yearly proportion of high-priority major uro-oncological surgeries at three Italian high-volume referral centers was done. This review measured the burden of nondeferrable major uro-oncological surgery to build a strategy of selection during the COVID-19 pandemic. Overall, 2,387 patients were included. Of these, 771 (32.3%) were classified as a high priority. Stratifying these surgeries by cancer type, radical nephroureterectomy, nephrectomy, radical prostatectomy, radical cystectomy accounted for 12.6, 17.3, 33.9, and 36.2%, respectively, of high-priority procedures. It is notable that a non-negligible proportion of patients undergoing high-priority major cancer surgeries are at higher perioperative risk that may need postoperative surveillance in the intensive care unit. 25

A survey involving 57 European urological referral centers was conducted by Oderda et al. Results showed that that the management of the main urological cancers has been altered dramatically by the COVID-19 pandemic, with most European centers (82%) declaring to be “much” or “very much” affected. Uro-oncological consultations for newly diagnosed cancers and follow-up were more than halved or almost suspended, in 55 and 71% of centers, respectively. In March 2020, a dramatic reduction was seen in major uro-oncological surgeries across Europe: radical prostatectomies, radical cystectomies, radical/partial nephrectomies, and nephroureterectomies decreased by 53, 41, 53, and 52%, respectively. The majority of radical prostatectomies nowadays are performed with the robotic technique at referral centers, but the restrictions have specifically affected the access to the robot. 26 Another survey was done to assess how Italian oncologist reacts to the shifting of genitourinary oncology during the COVID-19 pandemic. A total of 72 physicians provided feedback. In general, there was consensus among oncologists to pursue treatment, possibly without delays or interruptions, for patients with locally advanced or metastatic disease for which an induction or first-line therapy option is indicated in guidelines. For advanced disease without curative intent, a non-negligible number of oncologists would delay treatment initiation. 27

Prostate cancer is one of the most common cancers that are treated by radiation oncologists. Ultrahypofractionation was preferred for localized, oligometastatic, and low volume M1 disease and moderate hypofractionation was preferred for post-prostatectomy and clinical node-positive disease. Salvage was preferred to adjuvant radiation. Patients with unfavorable intermediate, high, very high-risk, post-prostatectomy, clinical node-positive, oligometastatic, and low volume M1 can variably delay in-person new patient consultations, and return visits, but these should be converted to timely remote telehealth visits. In the COVID-19 pandemic, the shortest safe regimen can be used. 28 Man with advanced prostate cancer is generally older, frail, and has multiple comorbidities. For patients with nonmetastatic disease and a PSA doubling time of >9 months, initiating ADT may be suspended. Systemic treatment should be offered to patients with hormone-sensitive M1 disease; the standard of care would be ADT  +  androgen receptor axis targeted therapy (ARAT). Docetaxel should be avoided. 29 EAU considers the treatment for metastatic castration-resistant (mCRPC) as a high priority. Thus, treatment with life-prolonging agents should be initiated in <6 weeks. 30 As the first line therapy for mCRPC, ARATs are recommended when these therapies have not been used previously. If ARATs have been used previously (in the nonmetastatic-CRPC or mHSPC [hormone-sensitive] settings) chemotherapy would be a next-line recommendation. Glucocorticoids should be minimized as an adjunct to systemic therapies in an escalating COVID-19 pandemic. 31 Most immunotherapies for different prostate cancer patient subpopulations with advanced disease will be given as a part of a clinical trial and will be suspended during the SARS-CoV-2 pandemic. 32 Data has been showed that ADT might protect men from COVID-19 infection. A mechanistic explanation for this theory is related to the viral entry mediated by transmembrane serine protease 2 (TMPRSS2). 33 ADT may have a protective effect in decreasing the severity of COVID-19. 34

For the patient with bladder cancer undergoing treatment, there are several safety issues that place them at higher risk of infection for COVID-19 compared with the general population. A meta-analysis of 13 studies suggested that a delay of more than 12 weeks from the time of diagnosis to execution of radical cystectomy, only in muscle invasive urothelial cancer, was associated with worse outcomes. 35 High priority cases should be treated within <6 weeks and include: TURBT for suspicious invasive tumor identified on imaging; consider alternatives such as radiotherapy ± chemotherapy to palliative radical cystectomy; neoadjuvant chemotherapy (NAC) for individualize risk in high burden T3–4 N0M0 patients while they are on the waiting list; offer adjuvant cisplatin-based combination chemotherapy to patients with T3–4 and/or pN+ disease if no NAC was given. 36 High-risk nonmuscle-invasive bladder cancer (NMIBC) progression and worse prognosis are also characterized by a higher incidence in patients with risk factors similar to COVID-19. Immune system response and inflammation have been found as a common hallmark of both diseases. 37 A hemostatic radiotherapy is an option for actively bleeding MIBC when radical cystectomy is deferred due to limited resources. 38

Radical orchiectomy should be performed as soon as possible because it is an outpatient procedure and will guide further treatment. For low-volume stage II patients (IIa and IIb), radiotherapy instead of chemotherapy for seminomas is recommended. For high volume stage II seminomas: four cycles of etoposide and cisplatin is recommended. 39 Chemotherapy should be withheld until an active COVID-19 infection has resolved or has been ruled out with highly accurate molecular testing. Because of lacking data on increased lung toxicity in case of COVID-19 infection, bleomycin should not routinely be omitted. 40

During the COVID-19 pandemic, kidney cancer scenarios are quite different depending on their stage, distinguishing mainly between the low priority of localized disease or high priority of locally advanced and metastatic under active treatment. 41 Patients with cT1a kidney tumors must be put under active surveillance and delayed intervention. cT1b-T2a/b tumors must be managed by partial or radical nephrectomy, some selected T1b-T2a ((≤7 cm) cases can have the surgery postponed. 42

Urologists should provide complete clinical information with each specimen to a pathologist if possible and to refrain from submitting fresh-frozen sections for evaluation during the COVID-19 pandemic. 43

## Systemic Therapy and Intravesical Instillation of Bacillus Calmette-Guérin for Urologic Cancer Patients during COVID-19 Pandemic

Adjuvant and neoadjuvant treatments require particular attention. The risk/benefit ratio may favor not giving therapy if the survival benefits are modest or unproven, such as perioperative therapy in urothelial cancer. For prostate cancer, chemotherapy should not be commenced without justification for a patient at a significant risk of COVID-19. ADT should not be stopped without justification. For renal cell carcinoma, immune checkpoint inhibitor or oral vascular endothelial growth factor targeted therapy after a prolonged period (1–2 years) can be stopped or delayed. For urothelial carcinoma, chemotherapy for platinum-refractory patients who are not responding to therapy can be stopped or delayed. For germ cell tumor, first- and second-line treatment for metastatic disease should not be stopped without justification. 44

There is an overlap between the coronavirus-related interstitial pneumonia and the possible lung toxicity from anti-PD-1/PD-L1 agents. Immune checkpoint inhibitors-related pneumonitis ranges from 2.5 to 5%. Immunotherapy in the current context of the COVID-19 pandemic should be characterized by separated reflections. 45

The daily incidence of COVID-19 was 0.8 per million in countries with Bacillus Calmette-Guérin (BCG) vaccination compared with 34.8 per million in countries without such a program. Intravesical BCG should be continued during the COVID-19 pandemic because it remains the gold-standard adjuvant treatment for patients with high-risk NMIBC to prevent recurrence and progression. There was no data that mention if patients receiving intravesical BCG have a higher risk of contracting COVID-19. 46 In the absence of COVID-19, six weekly doses of BCG induction should be completed for high-risk NMIBC patients. Concerning the maintenance regimen, giving of at least two out of the three doses of a BCG maintenance course should be considered acceptable. BCG can be stopped safely for patients who are taking already BCG for 1 year. In the presence of confirmed COVID-19, a cautionary approach would be to delay instillation of BCG given that there are no data on tolerance of intravesical BCG among COVID-19 patient. Recent theories, that suggest the use of BCG as a vaccination which could prevent COVID-19, need to be approved. 47 Intravesical BCG should be continued during the COVID-19 pandemic because, to date, there are no reports that patients receiving intravesical BCG have a higher risk of contracting COVID-19. 48 However, the increased risk of COVID-19 transmission should be kept in mind during intravesical BCG therapy and protective measures against COVID-19 for health care providers and patients before the procedure should be taken optimally. 49

Case numbers of COVID-19 per million people in countries with a national BCG vaccination program were statistically significantly lower than those that did not have or have ceased their national BCG vaccination programs. Until a specific vaccine is developed for coronavirus, vulnerable populations could be immunized with BCG vaccines. 50

In the context of cancer care, there is a dilemma amongst oncologists about whether to use corticosteroids as part of anticancer regimens, supportive care, and toxicity management. There is currently no evidence that corticosteroid therapy in cancer patients increases the risk of infection with COVID-19. 51

## Minimally Invasive Surgery during COVID-19 Pandemic

Among surgeons worldwide, a concern with the use of minimally invasive techniques has been raised due to a proposed risk of viral transmission of the COVID-19 with the creation of pneumoperitoneum. The use of laparoscopy during the pandemic can contribute to decrease the length of stay as compared with open surgery as well as minimizing the need for medical treatments, and in turn increasing availability of beds, a limited resource. To decrease the risk of transmission of COVID-19 during laparoscopy, it is recommended to ensure a closed circuit for insufflation with the use of some sort of smoke evacuator device to avoid any release of pneumoperitoneum into the room, if no smoke evacuator systems are available, direct suction to laparoscopic trocars can be used. It is recommended also to reduce pneumoperitoneum pressures. Additional precautions to take with robotic surgery to avoid leakage from trocars include always using the trocar reducers in 12-mm trocars when inserting 8-mm or 5-mm instruments through the 12-mm trocars. 52 The release of aerosol through the trocar valves might potentially expose the OR staff to the coronavirus. Levels of pneumoperitoneum pressure and the power settings of electrocautery should be as low as possible to reduce possible aerosol formation. The OR staff needs substantial protection during all procedures and in particular, during minimally invasive surgery (MIS). 53 Also, the need to use appropriate PPE should be reinforced. Nasopharyngeal samples should be considered for all patients undergoing such procedures, especially as COVID-19 positivity could have a possible impact on their postoperative course. 54

The evidence against MIS appears not to be balanced with its proven benefits. In particular, the lower morbidity and short stay in the hospital that characterize MIS appear to be clear advantages in a period when health care systems are stressed to the limits. Blood losses are lower in MIS compared with open surgery and this aspect is particularly important in a period where blood supplies are lacking, due to self-isolation and quarantine. 55 In fact, pure laparoscopic surgery, or robot-assisted, seems to be safer, favoring both patients and the professional team that assist them. Although the risk of exposure to aerosols appears to be higher in MIS than in open surgery, the latter has an extremely higher risk of spreading micro and macroparticles, blood, and tissues to the surgical team. 56 The Surgical societies recommend that there is no clear evidence of COVID-19 virus in the CO _2_ used during MIS procedures or in the plume created by electrocautery. Until the presence or absence of COVID-19 viral particles have been clearly established; measures to mitigate CO _2_ should be taken and surgical cautery plume release into the OR should be performed. 57

There are many reports of coronavirus viability in aerosols. These modes of transmission may affect the safety to perform minimally invasive surgeries. All patients about to undergo those type of surgeries will need to be screened for symptoms and exposure.

## Endourology during COVID-19 Pandemic

The persistence and clearance of viral RNA from different specimens of patients with COVID-19 remain unclear. Results for only 6.9% of urine samples of patients were positive for viral nucleic acid in a study done by Ling et al. 58

It is recommended that endoscopic procedures and urethral catheterization should be performed with caution and the surgeons should be completely protected against infection if the patient has a suspicious or confirmed COVID-19 disease. 59 In the case of an obstructed/infected kidney, only decompression of the system is suggested, which can be achieved safely via either stenting or percutaneous nephrostomy. Whenever possible, the ureteral stent or nephrostomy tube should be placed under local anesthesia, sparing a ventilator. 60 In a survey, urologists treated more patients with complicated urolithiasis such as patients with acute renal failure and pyelonephritis and only 6.4% of the respondents responded that the approach to stone patients in the emergency department is the same as before. 61 Urinary stone emergencies are mainly severe; their care needs to be continued and they were not significantly influenced by the COVID-19 pandemic. 62 Suggested details to help the selection of endourologic procedures during the COVID-19 pandemic are summarized in Table 3 .

**Table 3:** Details of the high priority endourology procedures during the COVID-19 pandemic

High priority surgery	Details
Percutaneous nephrostomy or JJ stent insertion	Obstructed kidney ± infection Solitary kidney Bilateral ureteral obstruction Symptomatic ureteral stone failed on MET
TUR	Control of bleeding.

Abbreviations: MET, medical expulsive therapy; TUR, transurethral resection.

NSAIDs (nonsteroidal anti-inflammatory drugs) provide optimal analgesia in renal colic due to the reduction in glomerular filtration and renal pelvic pressure, ureteric peristalsis, and ureteric edema. 63 The existing literature does not currently provide conclusive evidence for or against the use of NSAIDs in the treatment of COVID-19 patients. 64

## Kidney Transplantation during COVID-19 Pandemic

About transplant procedures, SARS-CoV-2 infection could be missed in both donors and recipients who are asymptomatic owing to the sensitivity issues with the reverse transcription-polymerase chain reaction test. There is insufficient evidence to consider kidney transplantation as a safe procedure in COVID-19 pandemic areas. Decisions should be made on a case-by-case basis according to the patient situation. 65 Gandolfini et al reported the outcomes of two deceased-donor kidney transplant recipients with COVID-19 pneumonia. In the presented kidney transplant recipients, the course of COVID-19 did not significantly differ from that of nontransplant individuals. Immunosuppression interruption combined with the anti-inflammatory effects of colchicine may have synergized with antiviral therapy and hydroxychloroquine to lower viral replication and minimize the cytokine storm triggered by SARS-CoV-2. 66 Banerjee et al presented seven cases of COVID-19 infection in kidney transplant recipients from south London, United Kingdom. Of seven patients, two were managed on an outpatient basis and stayed at home, with the remaining five requiring hospital admission. Four among the latter required intensive care unit admission, and one is being managed in the renal ward. The mortality rate of their series was 14%. Managing immunosuppression in these patients is challenging. In transplant patients with mild to moderate infections, the usual practice is to continue or make reductions in the dose of immunosuppressive drugs. In severe infections (requiring intubation and ventilation), an argument can be made for stopping calcineurin inhibitors completely while maintaining corticosteroid therapy. 67 It is recommended to suspend kidney transplantation during the COVID-19 pandemic especially for high-risk older recipients with comorbidities.

Due to a lack of evidence, the immunosuppressive strategy in kidney transplant patients with COVID-19 is not clear yet. A protocol was suggested in hospital La Paz in Madrid, Spain depends on the age and the severity of the infection. For kidney transplant patients, less than 60 years old without lung infiltrate, immunosuppressive therapies were recommended and the dose of on hydroxychloroquine was adjusted. For patients without lung infiltrate, more than 60 years, it was recommended to suspend mycophenolate mofetil (MMF) and maintain with tacrolimus (levels 4–6 ng/mL) and prednisone (usual dose, do not go up to 20 mg). For patients with lung infiltrate, less than 60 years, without hypoxemia, it is recommended to discontinue MMF and maintain tacrolimus (levels 4–6 ng/mL) and prednisone 20 mg daily. If patients had hypoxemia, it is recommended to discontinue tacrolimus and MMF and maintain only with prednisone 20 mg a day for the first 4 days. For patients with lung infiltrate more than 60 years with hypoxemia, it is recommended to discontinue tacrolimus and MMF and maintain only with prednisone 20 mg a day for the first 4 days. 68

In the series of clinical cases reported by Alberici et al, all patients had baseline immunosuppression withdrawn. The clinical outcomes for the transplant patients were poor with 25% mortality mainly due to complications from pneumonia. Unfortunately, many confounding and selection biases, not least of which is the small sample size in reports by Alberici et al and Banerjee et al, do not allow us to draw firm conclusions from these fascinating first experiences. 69

There is uncertainty about the risks of transmission from the use of COVID-19-positive donors. There is a prioritization of intensive care capacity for COVID-19 patients and thus restricted availability for the care of both donors and postoperative nonrenal transplant recipients. 70 The routine use of corticosteroids to treat patients with SARS-CoV-2 is not recommended. Renal transplant recipients with moderate oxygen requirements may be able to be managed successfully with steroid-sparing modifications to immunosuppression including modest reductions in calcineurin inhibitor trough concentrations and antiproliferative dosing. 71 Mild-to-moderate symptomatic kidney transplantation recipients can be managed in an outpatient setting, while patients exhibiting severe symptoms must be admitted to the hospital. More rapid clinical progression, and higher complication and death rates have been observed for hospitalized kidney transplantation recipients, requiring hemodialysis or ventilatory support. Lymphopenia, elevated serum markers (C-reactive protein, procalcitonin, IL-6, D-dimer), and chest X-ray findings consistent with pneumonia are linked to a worse prognosis. 72

## Teleconferencing and Telemedicine in Urology during COVID-19 Pandemic

The 2020 annual meeting of the European Association of Urology due to be held in July has been postponed. The annual meeting of the American Urological Association has also been canceled and will be replaced by a virtual education symposium. The most modern tool for live distance learning is webinar technology. All the scientific content of the meeting should be transmitted online via real-time or delayed streaming. 73 Telemedicine consultations are effective in promoting social distancing and thus allow a significant proportion of patients to remain under home care. As a part of general plans in four hospitals in Brooklyn, New York, a decrease in outpatient services via utilization of telemedicine was implemented. 74 The imperative for most urology practices across the United States should be to convert almost all urology clinic visits to telemedicine visits or postpone the appointment. Performing those telemedicine visits decreased the financial burden on urology practice during the pandemic. 75

Many urological patients have risk factors associated with poor outcome from COVID-19 and most preferred telemedicine consultations at this time. This appears to be a solution to offer contact-free continuity of care. 76 Oncological follow-up of kidney, bladder, or prostate cancer could benefit from teleconsultations alternating with face-to-face encounters. 77

## Conclusion

The current COVID-19 pandemic underlines the importance of changing some aspects of urology practice, from patient consultation to the triage of urologic surgeries. The COVID-19 pandemic will lead to new genitourinary disease treatment guidelines. All the time urologists must prioritize the safety of their patients and staff. Telemedicine and teleconferencing can be used in this critical situation.
